# Esmolol Is Not the Solution: Thyroid Storm With Atrial Fibrillation

**DOI:** 10.7759/cureus.35201

**Published:** 2023-02-20

**Authors:** Zaid N Herzallah, Shreya Gupta, Maryam D Abdulhamid, Omar Q Muhammed Noori

**Affiliations:** 1 Emergency Medicine, Rashid Hospital Trauma Center/Dubai Health Authority (DHA), Dubai, ARE; 2 Internal Medicine, Rashid Hospital Trauma Center/Dubai Health Authority (DHA), Dubai, ARE

**Keywords:** borderline low blood pressure, atrial fib, heart failure, esmolol, thyroid-storm

## Abstract

Thyroid storm is a challenging medical emergency that requires urgent assessment and management in a timely manner. In this article, we report on a case of a 37-year-old female who presented to the emergency department with a thyroid storm complicated by atrial fibrillation (AF) with a rapid ventricular response with no clinical signs of heart failure. As part of her medical management to rate control her AF, she was started on an infusion of a short-acting beta blocker, esmolol, and shortly after, she developed cardiac arrest. This is the second case report published to highlight the significant response (cardiac arrest) of patients with thyroid storm complicated by AF to a low dose esmolol infusion as part of their medical management.

## Introduction

Thyroid storm is a life-threatening condition that is rarely seen in the emergency department. It occurs in 1-2% of hyperthyroid patients and it mainly presents in females who are in their third to sixth decade of life [1-9]. Patients with thyroid storm can present with variable symptoms: tachycardia, tachypnea, palpitation, and different types of arrhythmias like atrial fibrillation (AF), supraventricular tachycardia, and ventricular tachycardia. However, supraventricular tachycardia, flutter, and ventricular tachycardia are uncommon [10]. The medical treatment of AF seen in thyroid storm can be achieved by a rate control medication like a beta blocker. Propranolol is the preferred agent for beta-blockade in hyperthyroidism and thyroid storms due to its additional effect of blocking the peripheral conversion of inactive T4 to active form T3 [3]. Our patient presented with a history suggestive of thyroid storm and AF with borderline blood pressure. A decision was made to start managing the AF with a rate control medication, and esmolol was chosen over propranolol due to its short half-life.

## Case presentation

A previously healthy 37-year-old female of Asian origin presented to the Emergency Department (ED) via ambulance with palpitations for five days along with a one-day history of dyspnea, vomiting, and diarrhea. According to her relative, the patient had lost a significant amount of weight over the past year with heat intolerance and irregularities in her menstrual cycle.

**Figure 1:**
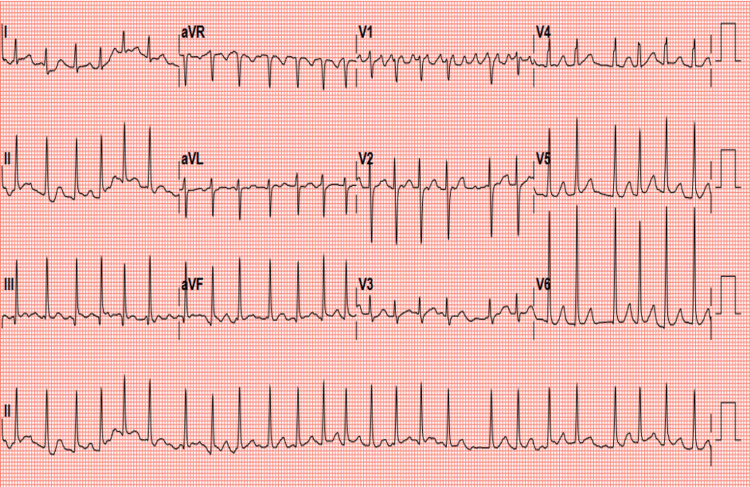
Electrocardiogram illustrates atrial fibrillation with rapid ventricular response

On examination, she was afebrile with a heart rate of 170-180 beats per minute (bpm), irregular, blood pressure of 104/64 mmHg, respiratory rate of 18 breaths per minute, and oxygen saturation of 98% on non-rebreather oxygen support. She was awake and oriented with Glasgow Coma Scale (GCS) 15/15, non-icteric and non-cyanotic. A neck examination revealed a thyroid swelling involving both lobes, soft, and not tender to touch. No clinical signs of heart failure were noted, and the rest of the physical exam was unremarkable. Point-of-care glucose testing was normal (107mg/dl). An electrocardiogram (Figure 1) was done within 10 minutes of arrival at the ED and showed AF with rapid ventricular response.

Based on these findings, the patient’s Burch-Wartofsky score was 55 points (temperature: < 99F, CNS: mild, gastrointestinal dysfunction: moderate, heart rate >140, congestive heart failure (CHF): absent, AF: yes, precipitating event: no), highly suggestive of thyroid storm. We decided to start the patient on an esmolol infusion at a rate of 50 mcg/kg per minute based on the presence of AF and no clinical signs of heart failure. The infusion was started without a bolus in view of her borderline blood pressure. Hydrocortisone 100 mg and intravenous fluids 500 ml of normal saline were also administered. Fifteen minutes after starting esmolol, the patient became restless and started complaining of difficulty in breathing. Within the next five minutes, despite escalating oxygen support, her heart rate significantly dropped to 40 bpm and she went into cardiac arrest, and the initial rhythm was asystole.

Advanced Cardiovascular Life Support (ACLS) protocol for cardiopulmonary arrest was initiated immediately. She underwent three cycles of cardiopulmonary resuscitation (CPR) until the return of spontaneous circulation (ROSC) was achieved. On assessment, her blood pressure had normalized to 109/78, and her GCS was 3/15, prompting a need for endotracheal intubation. She was placed on norepinephrine infusion and ACLS post-cardiac arrest care protocol was started.

Table 1 depicts the initial laboratory investigations. The results were indicative of high anion gap metabolic acidosis with a lactate level of 12.5 mmol/L on venous blood gas (VBG), thyroid stimulating hormone <0.005, free T4 >100, high white blood cell count with a high absolute lymphocyte count, anemia, elevated troponin T and brain natriuretic peptide, deranged liver function tests, and mild hyponatremia with hypochloremia.

**Table 1:** Pertinent results from laboratory investigations

Laboratory Investigation		Result	Laboratory Investigation		Result
Full Blood Count	White Blood Cell Count (10^3^/uL)	11.8 (H)	Thyroid Function Tests	Free T4 (pmol/L)	>100 (H)
	Neutrophil count (10^3^/uL)	6.6		Thyroid Stimulating Hormone (ulU/mL)	<0.005(L)
	Lymphocyte count (10^3^/uL)	4.5 (H)	Liver Function Tests	Aspartate Aminotransferase (U/L)	37 (H)
	Hemoglobin (g/dL)	9.9 (L)		Alanine Transaminase (U/L)	23
	Platelet (10^3^/uL)	404		Alkaline Phosphatase (U/L)	205 (H)
C-reactive Protein (mg/L)		34.7 (H)		Bilirubin total (mg/dL)	1.4 (H)
Serum K (mmol/L)		4.9		Albumin (g/dL)	3.6
Cardiac Biomarkers	Troponin T (ng/L)	16 (H)	Coagulation	Prothrombin Time (s)	29.4(H)
	NT-proB-type Natriuretic Peptide (pg/mL)	3046 (H)		International Normalized Ratio	2.87(H)

**Figure 2:**
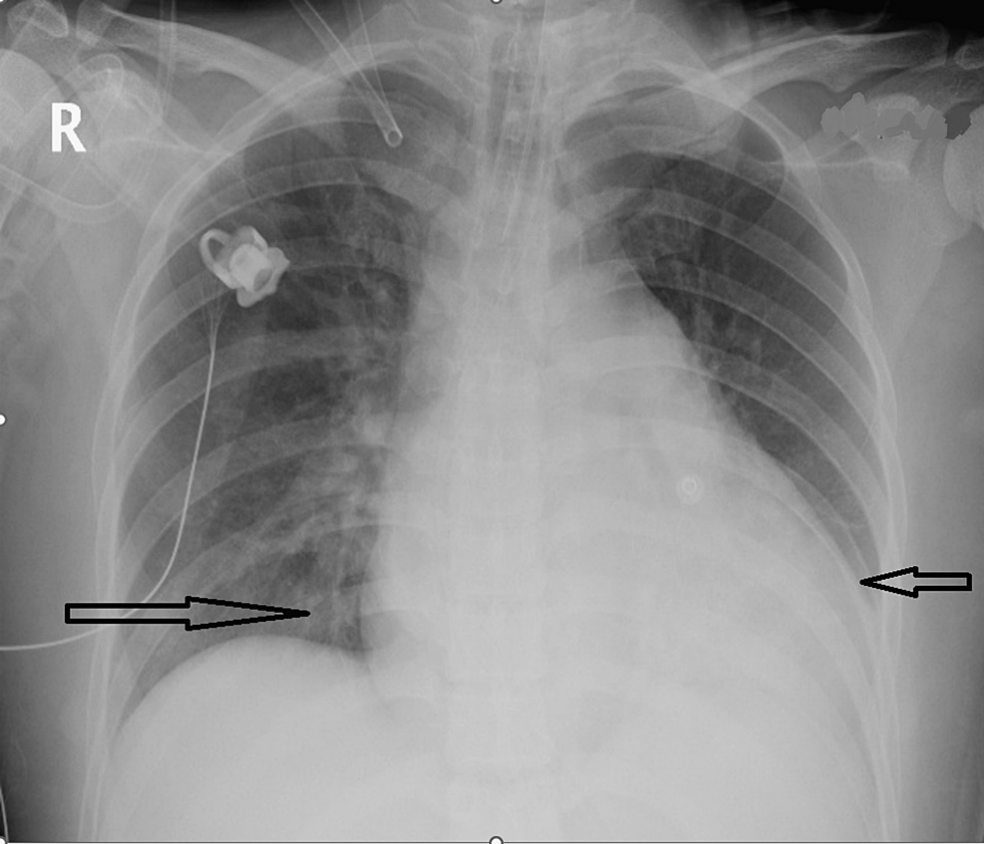
Chest x-ray showing cardiomegaly and patchy consolidations bilaterally in the mid and lower lung fields

Post-arrest CT brain and CT angiography were negative for acute brain pathologies but incidentally showed vasculitic changes in the extra-cranial internal carotid arteries bilaterally. Additionally, cardiomegaly with bilateral patchy mid and lower zone consolidations were noted on chest x-ray (Figure 2). A bedside echocardiogram, which was done later after admission by the cardiology team, showed a reduced left ventricular ejection fraction of 40%, moderate-to-severe mitral regurgitation, and dilated cardiomyopathy.

Once stabilized, the patient was admitted to the intensive care unit (ICU) and managed with propranolol, carbimazole, hydrocortisone, intravenous antibiotics, and continuous renal replacement therapy. Unfortunately, her clinical condition continued to deteriorate as she developed multi-organ failure, diffuse hypoxic brain injury, and eventual death.

## Discussion

The overall mortality of thyroid storm ranges between 10% and 30%, but in cases where there is a delay in initiating the treatment, it can increase up to 75% [1]. Arrhythmias like AF occur in up to 15% of patients with hyperthyroidism and it becomes more common in elderly male patients [5]. In a large study of patients with new-onset AF, less than 1% of AF incidence was caused by overt hyperthyroidism. Therefore, although serum thyroid-stimulating hormone (TSH) is measured in a large number of patients with new-onset AF to rule out thyroid disease, this association is uncommon in the absence of additional symptoms and signs of hyperthyroidism [6,8]. There are several ways hyperthyroidism can affect the cardiovascular system. Initially, the increase in thyroid hormone in the blood circulation will increase the expression of adenosine triphosphate (ATP), which will cause an increase in myocardial heart rate (chronotropy) and contractility (inotropy) and the result of this is an increase in the left ventricular ejection fraction and cardiac output [7,11]. Increasing the level of T3 will increase the hypermetabolic state of the body, which will increase the consumption of oxygen, increase the production of lactic acid, and will cause arterial smooth muscle relaxation. The systemic vascular resistance will also decrease consequently, causing a decrease in renal perfusion, which will lead to the activation of the renin-angiotensin system as a counter mechanism; this will lead to the expansion of blood volume [2,10]. Furthermore, this will result in a decrease in the afterload and an increase in the preload, which will lead to high-output heart failure. Once the heart becomes overwhelmed due to an increase in the workload, this may lead to a state of low-output heart failure.

The diagnosis of thyroid storm is mainly made based on clinical assessment (Burch-Wartofsky score) as the measurements of thyroid hormones will not help in differentiating between thyroid storm and hyperthyroidism. The main systems involved are the nervous systems with the following symptoms: confusion, altered mental status, and seizures. Palpitations, tachycardia, and AF are the main symptoms affecting the cardiovascular system, which can lead to heart failure and cardiovascular collapse. In the gastrointestinal system, the main symptoms are nausea, diarrhea, and vomiting [4,12]

The medical management of thyroid storm includes starting the patient on beta-blockade to lower the heart rate in cases of sinus tachycardia and AF. Propranolol has the additional effect to decrease the peripheral conversion of T4 to T3. Furthermore, thionamides (anti-thyroid medication) should be initiated to stop the synthesis of new thyroid hormones, then potassium iodide can be added to prevent the release of the performed hormone from the thyroid gland. Moreover, treating the patient with glucocorticoids helps stop the conversion of T4 to T3 [4]. In our case, with thyroid storm complicated by AF with no clinical signs of heart failure, we decided to start the patient on esmolol instead of propranolol due to its short half-life (half-life of nine minutes) in comparison to propranolol (half-life of three hours), considering that our patient’s blood pressure was on the lower side. Despite that, the patient rapidly deteriorated and ended up with a cardiac arrest. This raised the concern of subclinical heart failure for cases that were significantly affected by the introduction of a short-acting beta blocker leading to cardiopulmonary arrest.

There have been multiple reported cases of patients with thyroid storm who have collapsed and suffered a cardiac arrest shortly after starting propranolol [13-15]. Pong et al. [12], Dalan and Leow [14], and Modarresi et al. [16] clearly recommend starting esmolol for such cases due to the fact that it has a short life and is easily titratable. However, our report is the second [17] in the literature detailing sudden collapse and cardiac arrest after starting esmolol, and it clearly raises great concern over starting any beta blocker for patients with thyroid storm before a detailed clinical assessment for heart failure and, if available, a bedside echocardiogram to look for signs of low output heart failure [4]. If any of the above-mentioned is identified, it is better not to start the patient acutely on a beta blocker and focus on the management of the thyroid storm itself along with adequate hemodynamic support.

## Conclusions

This report highlights the development of cardiac arrest shortly after starting an esmolol infusion for a patient who presented with a thyroid storm complicated by AF. It is highly recommended not to start such patients on beta blockers if signs of heart failure are identified clinically or by an echocardiogram exam, but rather focus mainly on treating the thyrotoxicosis and hemodynamic support for the patient. Further studies are needed to look into the management of AF-complicated thyroid storm.
